# Autoantibodies targeting cytokines and connective tissue disease autoantigens are common in acute non-SARS-CoV-2 infections.

**DOI:** 10.21203/rs.3.rs-1233038/v1

**Published:** 2022-01-20

**Authors:** Allan Feng, Emily Yang, Andrew Moore, Shaurya Dhingra, Sarah Chang, Xihui Yin, Ruoxi Pi, Elisabeth Mack, Sara Völkel, Reinhard Geßner, Margrit Gundisch, Andreas Neubauer, Harald Renz, SOTIRIOS TSIODRAS, Paraskevi Fragkou, Adijat Asuni, Joseph Levitt, Jennifer Wilson, Michelle Leong, Jennifer Lumb, Rong Mao, Kassandra Pinedo, Jonasel Roque, Christopher Richards, Mikayla Stabile, Gayathri Swaminathan, Maria Salagianni, Vasiliki Triantafyllia, Wilhelm Bertrams, Catherine Blish, Jan Carette, Jennifer Frankovich, Eric Meffre, Kari C. Nadeau, Upinder Singh, Taia Wang, Eline Luning Prak, Susanne Herold, Evangelos Andreakos, Bernd Schmeck, Chrysanthi Skevaki, Angela Rogers, Paul Utz

**Affiliations:** Stanford University School of Medicine; Stanford University; Stanford University School of Medicine; Stanford University School of Medicine; Stanford University School of Medicine; Stanford University School of Medicine; University of Marburg; Philipps-Universität Marburg; University of Marburg; University of Marburg; University of Marburg; Philipps University Marburg; Hellenic Center for Disease Control; Attikon University Hospital; Stanford University School of Medicine; Stanford University School of Medicine; Stanford University; Stanford University School of Medicine; Stanford University; Stanford University; Stanford University School of Medicine; Stanford University; Stanford University School of Medicine; Stanford University School of Medicine; Stanford University School of Medicine; Academy of Athens; Biomedical Research Foundation Academy of Athens; Philipp University of Marburg; Stanford University; Stanford University School of Medicine; Stanford University; Yale University School of Medicine; Stanford University; Stanford University; Stanford University; University of Pennsylvania; a; Biomedical Research Foundation, Academy of Athens; Universitätsklinikum Gießen und Marburg; Institute of Laboratory Medicine; Stanford University; Stanford University School of Medicine

## Abstract

The widespread presence of autoantibodies in acute infection with severe acute respiratory syndrome coronavirus-2 (SARS-CoV-2) is increasingly recognized, but the prevalence of autoantibodies in infections with organisms other than SARS-CoV-2 has not yet been reported. We used protein arrays to profile IgG autoantibodies from 317 samples from 268 patients across a spectrum of non-SARS-CoV-2 infections, many of whom were critically ill with pneumonia. Anti-cytokine antibodies (ACA) were identified in > 50% of patients infected with non-SARS-CoV-2 viruses and other pathogens, including patients with pneumonia attributed to bacterial causes. In cell-based functional assays, some ACA blocked binding to surface receptors for type I interferons (Type I IFN), granulocyte-macrophage colony-stimulating factor (GM-CSF), and interleukin-6 (IL-6). Autoantibodies against traditional autoantigens associated with connective tissue diseases (CTDs) were also commonly observed in these cohorts, including newly-detected antibodies that emerged in longitudinal samples from patients infected with influenza. We conclude that autoantibodies, some of which are functionally active, may be much more prevalent than previously appreciated in patients who are symptomatically infected with diverse pathogens.

## Introduction

Infection is one of the most common reasons for health care visits. In the United States alone, infections accounted for an estimated 7 million encounters in physician offices and 3 million emergency room visits in 2018, and 8 billion dollars of direct health care costs in 2015^1–3^. A significant proportion of patients with severe infections are admitted to intensive care units (ICU) and develop acute respiratory distress syndrome (ARDS), which has a high mortality rate and leads to long-term morbidity in those who survive. The immunologic mechanisms underlying the development of severe infection and progression to ARDS are poorly understood^4,5^.

A substantial proportion of patients infected with severe acute respiratory syndrome coronavirus-2 (SARS-CoV-2) have serum autoantibodies that have been proposed to cause or contribute to clinical manifestations such as more severe respiratory failure, vasculitis, and thrombosis^6–9^. Over 60% of hospitalized coronavirus disease-19 (COVID-19) patients have one or more antibodies that recognize cytokines (anti-cytokine antibodies; ACA), some of which block binding and downstream signaling mediated through their cognate cell surface receptors^8,10,11^. We recently described newly-detectable IgG autoantibodies in COVID-19 patients, including ACA and antibodies to intracellular antigens associated with rare connective tissue diseases (CTDs), such as systemic sclerosis (SSc), myositis, and overlap syndromes^10^. Collectively, these studies suggest that pre-existing ACA, such as anti-Type I interferon (IFN), serve as permissive factors for severe COVID-19 in individuals with these antibody specificities, and that SARS-CoV-2 might trigger new CTD-associated autoantibodies (CTD-AAb) over time.

In addition to their clear role in COVID-19, ACA are known to be associated with several lung diseases, such as disseminated atypical mycobacterial infections (AMI, associated with anti-interferon-γ^12^) and pulmonary alveolar proteinosis (PAP, associated with anti-GM-CSF^13^). It is unknown whether ACA play a role in acute infections caused by other pathogens. We tested this hypothesis by screening for antibodies against cytokines and other autoantigens in blood samples from severely-ill patients who were either enrolled before the SARS-CoV-2 pandemic or were negative for SARS-CoV-2 by a PCR (polymerase chain reaction) test. Autoantibodies were more prevalent in patients admitted to the ICU with a known infection when compared with ICU patients who were thought to be uninfected. Although less prevalent, similar findings were observed in additional cohorts of patients with acute infection caused by influenza virus. Surprisingly, some patients with bacterial infections also had ACA capable of blocking receptor binding. Newly-detectable IgG autoantibodies were also discovered in some influenza patients, including antibodies specific for thyroperoxidase (TPO) and signal recognition particle 54 (SRP54), an autoantigen associated with myositis. Taken together, our studies suggest that autoimmunity is linked to not only SARS-CoV-2, but also additional viruses and potentially other classes of pathogens.

## Results

### Anti-cytokine autoantibodies (ACA) are highly prevalent in ICU patients, particularly those with infection.

**Figure 1:**
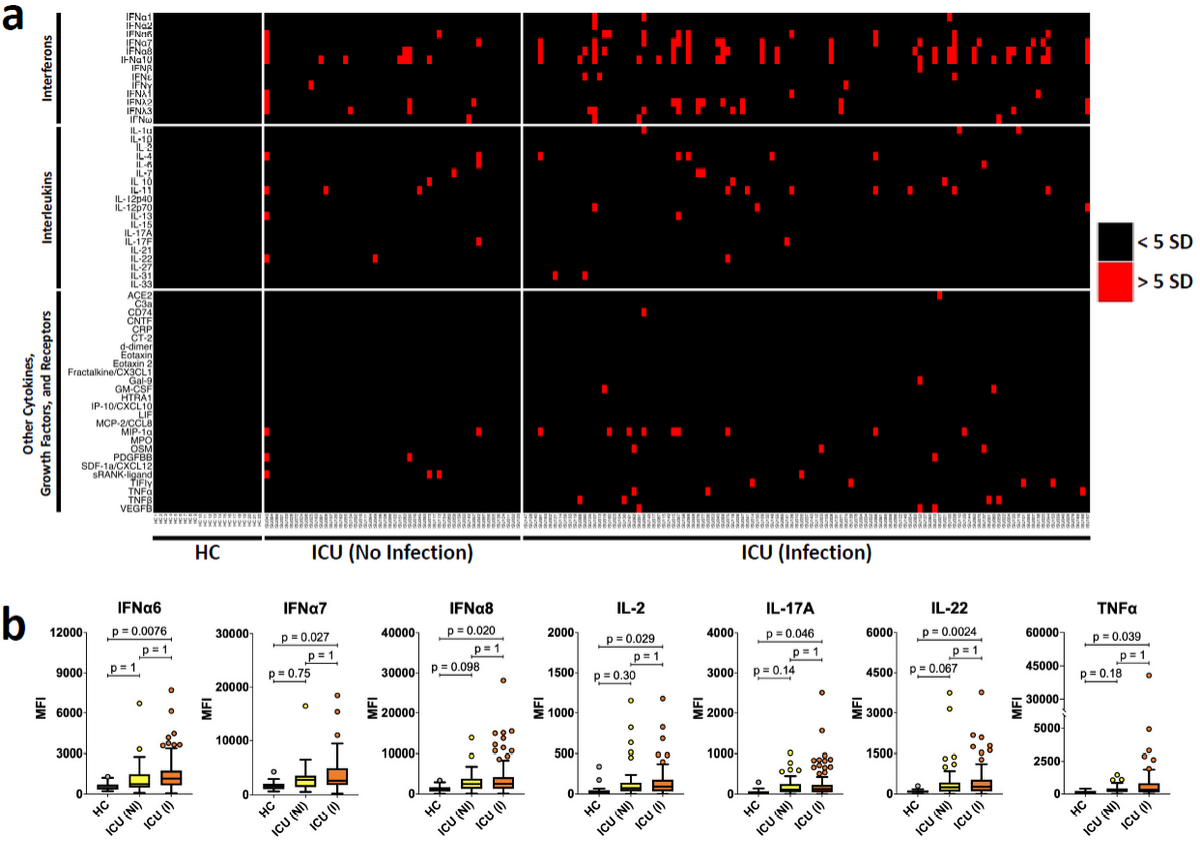
High prevalence of ACA in hospitalized ICU patients. **a** Heatmap representing serum IgG ACA discovered using a 58-plex array of cytokines, chemokines, growth factors, and receptors. Stanford ICU patients who were infected with viruses, bacteria, fungi, or a combination of pathogens (n = 115), Stanford ICU patients with no evidence for infection (n = 52), and HC (n = 22) were analyzed for ACA. Cytokines are grouped on the y-axis by category (interferons, interleukins, and other cytokines/growth factors/receptors). Colors indicate ACA whose MFI measurements are > 5 SD (red) or < 5 SD (black) above the average MFI for HC. MFIs <3,000 were excluded. **b** Tukey box plots comparing MFI data from HC and Stanford ICU patients for the seven antigens for which statistically significant differences were determined between patient groups using two-tailed Wilcoxon rank-sum tests with Bonferroni correction. The middle line represents the median, while the lower and upper hinges correspond to the first and third quartiles. The upper whisker extends from the hinge to 1.5 times the interquartile range (IQR) above the 75th percentile MFI value, and the lower whisker extends from the hinge to 1.5 times the IQR below the 25th percentile MFI value.

Using a custom 58-plex cytokine array (Supplementary Table 1), we screened for ACA in cross-sectional serum samples from 167 patients admitted to the Stanford ICU with at least one risk factor for ARDS (e.g., sepsis, aspiration, and/or trauma) and HC (Fig. 1). The clinical characteristics of the ICU cohort are shown in Supplementary Table 2 with 69% clinically phenotyped as infected, 59% with shock requiring vasopressors, and 25% with a 30-day mortality rate.

Based on the MFI criteria (5 SD > mean HC MFI and 3,000 MFI static cutoff) used in our recent COVID-19 study^10^, 84 of 167 (50%) cross-sectional ICU patient serum samples were positive for at least one ACA, while 0 of 22 HC were positive for ACA. Among the ACA-positive ICU patient samples, 39 were positive for a single cytokine, 39 for 2–5 cytokines, and 6 for six or more cytokines (Fig. 1a). Using two-tailed Wilcoxon rank sum tests with Bonferroni correction, we compared the ICU cohort with the HC group for each antigen in the array and identified 9 ACAs for which MFI levels in the ICU patient samples were statistically significantly higher than those of the HC samples (Supplementary Fig. 1). Using the Significance Analysis of Microarrays (SAM) algorithm, we identified 15 antigens with statistically significantly higher reactivity in the ICU patients compared to HC (Supplementary Fig. 2a). A subset of the statistically significant ACA cytokines (e.g., IFNα, IFNε, IL-22, and TNFα) have also been identified as common autoantibody targets in hospitalized COVID-19 patients^10^.

We next tested the hypothesis that autoantibodies, particularly ACA, were specifically associated with infection. We identified ICU patients who were admitted with a primary diagnosis of infection (n = 115, Fig. 1a, ***right panel***) and compared their autoantibody profiles with ICU patients classified as non-infected (n = 52, Fig. 1a, ***middle panel***). We found that 67 of 115 (58%) serum samples from infected ICU patients were positive for ACA targeting at least one antigen. In contrast, 17 of 52 (33%) serum samples from ICU patients thought to be uninfected were positive for ACA (OR 2.9, 1.4 – 6.1, p = 0.003). Although not statistically significant, we observed trends showing an association between autoantigen status and shock, and between autoantigen status and leukocytosis. Backwards stepwise logistic regression analysis evaluating sex, age, infection status, and APACHE score confirmed infection status as the only significant predictor of development of ACA (p = 0.003). Seven of the 9 previously identified statistically significant antigens, including IFNα6, IFNα7, IFNα8, IL-2, IL-17A, IL-22 and TNFα (Supplementary Fig. 1) were statistically significant when comparing the infection and HC groups (Fig. 1b).

### Autoantibodies that recognize traditional CTD antigens (CTD-AAb) are highly prevalent in critically ill patients, regardless of infection status.

Using the 5 SD > mean HC MFI and 3000 MFI cutoff criteria, we identified many CTD-autoantibodies (CTD-AAb), with 102 of 167 (61%) ICU patient serum samples positive for CTD-AAb recognizing at least one of 55 traditional CTD autoantigens in our array, particularly those associated with myositis (Supplementary Fig. 3). None of the HC samples (0 of 14) were positive for any of the 55 autoantigens. Autoantibodies recognized tRNA-synthetases PL-7 and EJ in 18 (11%) and 14 (8%) serum samples of 167 total, respectively, while MDA5 antibodies were identified in 13 subjects. Thyroperoxidase (TPO) was also commonly recognized by antibodies in 18 samples (11%). CTD-associated autoantigens (e.g., histones and PL-7) have also been identified as common autoantibody targets in hospitalized COVID-19 patients^10^. SAM analysis of the traditional autoantigen array data revealed that 10 of the 55 antigens had significantly higher MFI levels in the ICU cohort compared to HC (Supplementary Fig. 2b). Unlike with ACAs, there was no difference in CTD-AAb prevalence when comparing infected and uninfected ICU patients (data not shown). Autoantibodies were more common in older patients (66% of those > age 60 vs 50% of patients age <60, p = 0.04). Backwards stepwise logistic regression analysis evaluating sex, age, infection status, and APACHE score showed that age (p = 0.005) and APACHE II score (p = 0.04) were significant predictors of development of CTD-AAbs.

### Secreted proteins are recognized by autoantibodies in adult patients with acute influenza, and pneumonia attributed to other respiratory pathogens.

We screened serum samples from a cross-sectional cohort of patients from the University Hospitals of Giessen and Marburg with severe influenza (n = 25); a separate cohort of patients with ARDS (n = 17); patients admitted to the ICU in 2020 for COVID-19 symptoms, but who tested negative by PCR (n = 19); and HC (n = 11) using the 58-plex ACA array (Supplementary Fig. 4, ***upper panels***). Ten of 25 (40%) influenza patient serum samples recognized at least one of the 58 secreted or cell-surface antigens. Ten of 17 (59%) ARDS patient samples were positive for at least one ACA, and 9 of 19 (47%) COVID-19 negative patient samples were positive for at least one ACA. Autoantibodies specific for CTD antigens (CTD-AAb) were also observed in infected patients (Supplementary Fig. 4, ***lower panels***). In contrast, none of the HC were positive for autoantibodies directed against any secreted proteins or CTD autoantigens.

**Figure 2:**
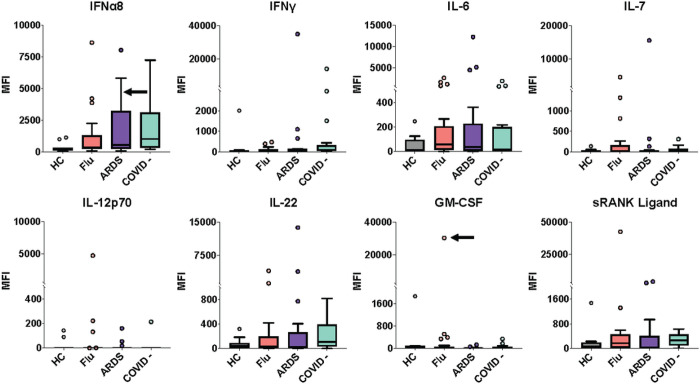
IgG anti-cytokine autoantibodies in serum from ARDS patients or patients acutely infected with influenza virus. Tukey box plots comparing MFI data for eight cytokines in influenza patients (n = 25) and ARDS patients (n = 17), both collected prior to the COVID-19 pandemic; ARDS patients who were COVID-19- (n = 19); and HC (n = 11). The middle line represents the median, while the lower and upper hinges correspond to the first and third quartiles. The upper whisker extends from the hinge to 1.5 times the interquartile range (IQR) above the 75th percentile MFI value, and the lower whisker extends from the hinge to 1.5 times the IQR below the 25th percentile MFI value. Black arrows indicate a serum sample with receptor blocking activity (see Fig. 3). Individual MFI values 1.5 times the IQR above the 75th percentile or 1.5 times the IQR below the 25th percentile are displayed as dots. MFI is shown on the y-axis, which is hatched to reflect outlier samples with relatively high MFI. Cohorts are shown on the x-axis.

Serum from a subset of influenza and ARDS patients had particularly high MFI ACA recognizing interferons, interleukins, and other cytokines (Fig. 2). The MFI level for IFNα8 in serum from one influenza patient was comparable to the IFNα8 MFI levels in prototype serum samples derived from two patients with Autoimmune Polyendocrinopathy Syndrome 1 (APS-1, MFI levels of approximately 8,000 and 7,000) which have receptor-blocking activity against IFNα8 in cell-based assays (Fig. 2)^14^. One COVID-19-negative patient sample and one ARDS patient sample also had comparable levels of anti-IFNα8 autoantibodies as the two APS-1 prototype serum samples (Fig. 2). Furthermore, the MFI level for anti-IFN-γ in one ARDS patient sample was comparable to that of the prototype serum sample derived from a patient with disseminated atypical mycobacterial infection (AMI, MFI level of approximately 26,000).

Striking reactivities against interleukins were observed for IL-6, IL-7, IL-12p70, and IL-22. Serum from one patient with influenza showed relatively high MFI levels of IgG specific for GM-CSF and soluble RANK-ligand. The MFI level of anti-GM-CSF autoantibodies in this sample was higher than in two pulmonary alveolar proteinosis (PAP) prototype serum samples (MFI levels of approximately 11,000 and 3,000). We conclude that patients with acute influenza or pneumonia caused by pathogens other than SARS-CoV-2 commonly have serum ACA.

### A subset of ACA has functional receptor blocking activity

**Figure 3:**
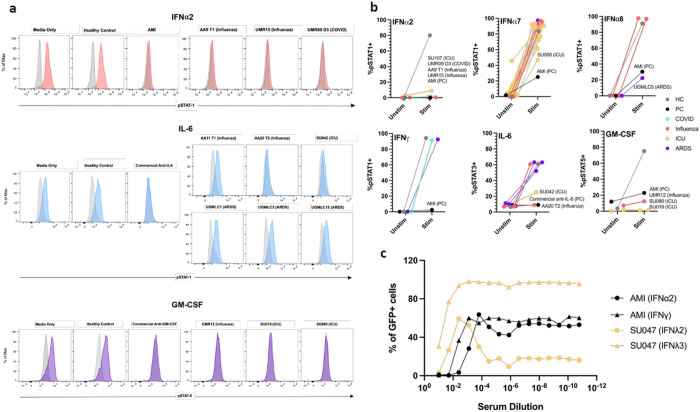
Cell-based cytokine receptor blocking assays. **a** Representative fluorescence-activated cell sorting (FACS) plots of IFNα2, IL-6, and GM-CSF signaling assays. Cells were treated with media only; commercial blocking antibody or 10% positive control serum from a patient with atypical mycobacterial infection (AMI); 10% healthy control serum; or 10% test serum. Cells were treated with patient serum or a control in the unstimulated condition (gray) and with both cytokine and patient serum or a control in the stimulated condition (red, blue, purple). **b** Blocking activity of patient serum on cells in cytokine signaling assays, reported as percentage of pSTAT positive cells in the unstimulated and stimulated condition. Patient sera were from COVID-19 (n = 2), influenza (n_Giessen/Marburg_ = 4, n_Athens_ = 5), Stanford ICU (n_infected_ = 19, n_non-infected_ = 2) and ARDS (n = 8) patients. For IFNα2 and IFNα8, results shown represent two independent experiments (Supplementary Fig. 5). Representative healthy controls (HC) and positive controls (PC: commercially available antibody or prototype patient serum with known blocking activity) are also included. **c** Neutralization activity to IFNα2, IFNγ, IFNλ2 and IFNλ3 in the serum samples of two patients. IFNα2, IFNγ, IFNλ2 and IFNλ3 were incubated with heat-inactivated serum from Donor AMI (positive control) and Donor SU047 (infected Stanford ICU cohort) and added to HAP1 reporter cells. The serum samples were prepared and tested with a 5-fold serial dilution on HAP1 reporter cells. Final concentrations of IFNα2, IFNγ, IFNλ2 and IFNλ3 in the culture were 40 U/ml, 8 U/ml, 1 ng/ml and 1 ng/ml, respectively. The percentages of GFP^+^ HAP1 reporter cells were evaluated 22–24 hours after the incubation with flow cytometry.

We used cell-based cytokine blocking assays to assess whether patient sera with high MFI values for ACA inhibit receptor-signaling in vitro (Fig. 3a). For each cytokine blocking assay, patient sera were selected based on an MFI value >5 SD above the healthy control average and a threshold of >3,000 MFI (Figs. 1 ***and*** 4, and Supplementary Fig. 5). A total of 39 samples met these criteria and were available for assessment of blocking activity (Stanford ICU, n_infected_ = 20, n_non-infected_ = 2; ARDS, n = 8; Giessen/Marburg influenza, n = 4; and Athens influenza, n = 5). We also analyzed two COVID patient sera we had previously identified^10^. Blocking activity was compared to that of a healthy control from Stanford Biobank, and either commercially available monoclonal receptor blocking antibodies or positive control prototype serum samples from patients with atypical mycobacterial infection.

ACA blocking activity for at least one cytokine was observed in 12 of 39 samples analyzed (31%, see Table 1 for summary and clinical characteristics). Using pSTAT signaling blocking assays for type I interferons, complete or partial blocking of STAT1 phosphorylation was observed in all 3 patient serum samples positive for anti-IFNα2 autoantibodies (Fig. 3a), 1 of 22 anti-IFNα7+ samples, and 1 of 3 anti-IFNα8+ samples (Fig. 3b, ***top 3 panels***). Complete or partial blocking of STAT phosphorylation was observed in 2 of 6 anti-IL-6+ samples and in all 3 anti-GM-CSF+ samples (Fig. 3, a and b). We also evaluated the blocking activity of a serum sample positive for anti-IFNλ2 and anti-IFNλ3 using a GFP reporter cell line that indicates IFN activation. Blocking of GFP expression was observed for both the IFNλ2 and IFNλ3 stimulating conditions (Fig. 3c).

Blocking activity was observed in at least one serum sample from all patient cohorts studied. Interestingly, of the 6 Stanford ICU serum samples that demonstrated blocking activity, 1 was derived from a patient with no signs of infection (SU042, Table 1) and 3 were derived from patients with laboratory-confirmed bacterial infections (SU107, SU047, and SU080, Table 1). Samples with blocking activity did not always correspond with the highest MFI values for the relevant ACA, which has been previously observed in COVID-19^32^. As reported in severely-ill COVID-19 patients, none of the anti-IFNγ+ samples had blocking activity, while samples from a patient with atypical mycobacterial infection had robust IFNγ receptor blocking activity (Fig. 3b, ***bottom left panel***). Thus, a fraction of ACA displays functional receptor blocking activity, whereas the relevance of other ACA remains unclear.

### Longitudinal profiling of CTD-AAbs identifies newly-detectable autoantibodies in patients with acute influenza infection.

**Figure 4:**
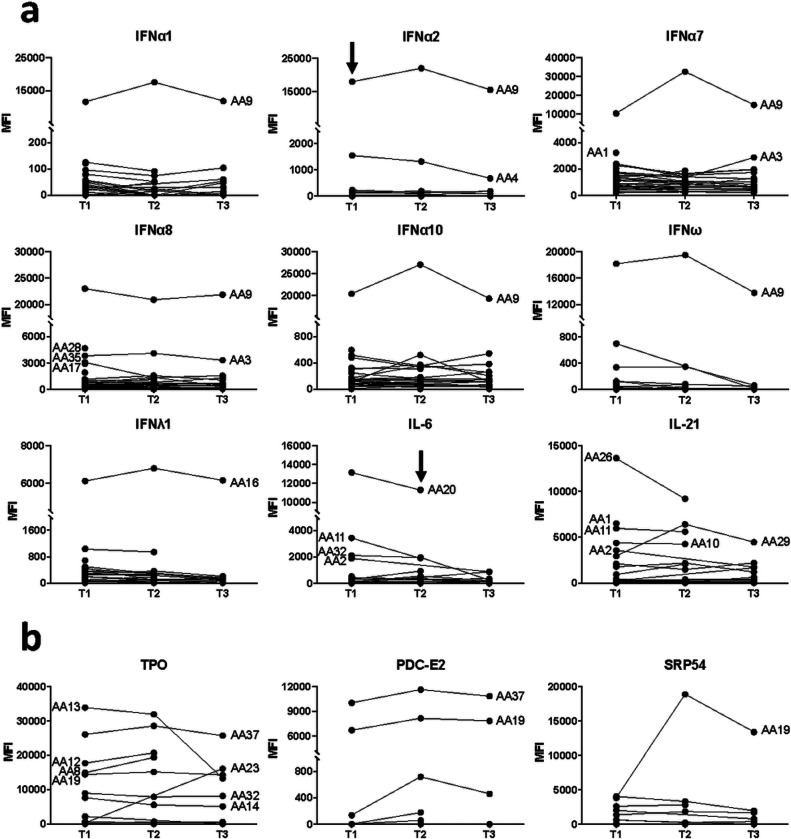
Newly-detectable autoantibodies in acutely-infected influenza patients. **a** Longitudinal measurements of specific anti-cytokine antibodies over time in acutely infected patients (n = 40). Serum was collected at 3 timepoints for 18 influenza subjects, at 2 timepoints for 13 influenza subjects, and at only the first timepoint for 9 subjects. The first timepoint (T1) is from the day that the patient was admitted to the hospital and diagnosed with influenza. T2 and T3 refer to approximately one week and one month, respectively, following hospital admission. MFI is shown on the y-axis, which is hatched to reflect outlier samples with relatively high MFI. Black arrows indicate a serum sample with receptor blocking activity (see Fig. 3). **b** Newly-detectable IgG autoantibodies recognize CTD autoantigens. Line plots display MFI levels of antibodies targeting traditional autoantigens that are inducible (SRP54 in subject AA19; TPO in subject AA23); fluctuate (TPO in subject AA13); or do not change significantly over time (most subjects with TPO autoantibodies, and two subjects with anti-PDC-E2).

To determine whether autoantibodies recognizing traditional autoantigens or cytokines were pre-existing versus newly-induced in response to viral infection, we screened a cohort of 40 influenza patients from the Biomedical Research Foundation of the Academy of Athens who came to medical attention for respiratory tract infections between December 2018 and April 2019. Serum was collected during the day of hospitalization (T1) for all 40 patients, then approximately one week later (T2) and approximately one month later (T3) for 29 and 20 patients, respectively. (Fig. 4, Supplementary Table 3).

One patient (Subject AA9) had autoantibodies in the baseline sample that targeted multiple IFNα subtypes (IFNα1, IFNα2, IFNα7, IFNα8, and IFNα10) as well as IFNω (Fig. 4a). Levels of anti-IFNα1, anti-IFNα2 (which had blocking activity (Fig. 3)), anti-IFNα8, anti-IFNα10, and anti-IFNω did not change significantly (MFI increase or decrease by 50% or more) across the three timepoints. In contrast, levels of antibodies recognizing IFNα7 fluctuated over time for Subject AA9. Relatively high and consistent levels of anti-IFNλ1 were observed in all three samples from Subject AA16. Multiple patients had relatively high levels of autoantibodies targeting IL-21 in their baseline samples (Fig. 4a).

We also measured levels of autoantibodies targeting traditional autoantigens and observed newly-detectable autoantibodies in serum samples from two patients (Fig. 4b). In Subject AA19, autoantibodies recognizing Signal Recognition Particle 54 (SRP54), a myositis-associated autoantigen, increased by over four-fold between the first and second timepoints, suggesting that autoantibodies were generated in response to influenza infection. Interestingly, the baseline serum sample from this patient also had autoantibodies against PDC-E2, a component of the mitochondrial pyruvate dehydrogenase complex that is associated with Primary Biliary Cirrhosis (PBC), and thyroperoxidase (TPO), a common autoantigen in autoimmune thyroid disease, suggesting Subject AA19 had underlying autoimmunity at the time of infection. MFI levels for both PDC-E2 and TPO did not change significantly over time for Subject AA19, and many other influenza patients also had anti-TPO that did not change over time. Anti-TPO MFI levels in another patient (Subject AA13) were consistently high at the first two timepoints (i.e., at the time of hospitalization and approximately one week later) but decreased significantly at the third time point (i.e., approximately a month after hospitalization), suggesting that anti-TPO may be transient. In contrast, Subject AA23 did not have detectable anti-TPO at baseline but had high MFI anti-TPO at the only other available timepoint approximately one month later. Taken together, these data suggest that a subset of patients acutely infected with influenza develop new autoantibodies that were undetectable in the baseline sample.

## Discussion

We recently demonstrated that ~25% of hospitalized patients infected with SARS-CoV-2 develop newly-detectable IgG autoantibodies that recognize cytokines and autoantigens typically associated with CTDs, such as myositis, SLE, and systemic sclerosis^10^. A critical unanswered question in these studies is whether autoantibodies are triggered in other acute infections, and if so, whether the target antigens differ from those identified in COVID-19. Here, we report that ACA and other antibodies known to be present in CTDs or previously identified in hospitalized COVID-19 patients are indeed found across a spectrum of patients with non-SARS-CoV-2 infections, including infections caused by viral pathogens and known or suspected bacterial pathogens. Pulmonary and non-pulmonary infections were also associated with ACAs, suggesting that secreted proteins are targeted across a spectrum of organ systems. Although the differences were not statistically different, autoantibodies specific for CTD antigens were more prominent in patients with infections than in those thought to be uninfected, and many autoantibodies were significantly more prevalent in these two groups than in healthy controls. Longitudinal data suggests that while most of these ACA are present at the time of presentation, some can emerge over time and can persist to at least 28 days after infection.

Anti-Type I interferon (Anti-Type I IFN) antibodies have been a major focus of ACA studies to date, particularly those on COVID-19^11,15–17^. Anti-Type I IFN are prevalent in severe COVID-19, particularly in men, but not in asymptomatic or mildly ill patients with SARS-CoV-2 infections. Multiple publications have demonstrated that a subset of anti-Type I IFN block binding to the interferon-α/β receptor (IFNAR), prevent activation of JAK-STAT signaling pathways, and facilitate viral replication in *in vitro*, cell-based models^8,14,15,18^. A causal link between pre-existing anti-Type I IFN and pathogenesis is suggested by the increased mortality observed in SARS-CoV-2-infected APS-1 patients,^17^ and poorly controlled infection in patients exposed to the live, attenuated yellow fever virus vaccine^16^. It remains unclear whether anti-Type I IFN remain as fixed components of the autoantibody repertoire of severely-ill COVID-19 patients and whether they might predispose such patients to superinfection or subsequent severe infection with other pathogens^19^. A recent study suggests that anti-Type I IFN in patients with APS-1, thymoma, or RAG-1 deficiency are long-lived while COVID-19-associated anti-Type I IFN levels decrease substantially over time, but with persistence of anti-Type I IFN with blocking activity^20^.

Here we show that anti-Type I IFN are also frequently found in ICU patients (Supplementary Fig. 1), particularly in patients with infections compared with those who appear to be uninfected (Fig. 1b). We identified 5 patients with anti-Type I IFN blocking activity (Table 1 and Fig. 3). Consistent with previous reports, all were over the age of 60, and 5 were male. A female patient (subject SU008) had IFNα7-neutralizing antibodies and developed ARDS following infection with respiratory syncytial virus (RSV). Subject UMR15 had IFNα2-neutralizing antibodies and developed severe influenza at age 66. Two years later, the same individual developed severe COVID-19 and ARDS, requiring mechanical ventilation two weeks after the first COVID mRNA vaccination and complicated by the reactivation of cytomegalovirus (CMV) and herpes simplex virus (HSV) pneumonitis. Although pre-infection samples are unavailable for other patients analyzed in this report, a subset of patients who develop symptomatic influenza or other respiratory infections most likely harbor pre-existing ACA that predispose them to develop ARDS when infected with more virulent pathogens, such as SARS-CoV-2. Because blocking ACA can be found even in patients with undetectable IgG autoantibodies by ELISA or bead-based assays used here, our results likely underestimate the true prevalence of functional ACA in patients with non-SARS-CoV-2 infections^15,20^. Future studies will be needed to understand the risk for superinfection as well as the efficacy of vaccines in patients with blocking ACA, including determining whether ACA such as anti-Type I IFN are enriched in patients with breakthrough influenza or SARS-CoV-2 infections.

Many other ACAs were identified in patients with infections other than SARS-CoV-2, including antibodies specific for interleukins (IL-2, IL-17A, and IL-22) and TNFα (Fig. 1b), and less frequent targets such as IFNγ, IL-6, IL-12p70, GM-CSF, and sRANK ligand (Fig. 2). We tested IgG-mediated receptor blocking activity for a handful of secreted proteins and identified at least one sample with IgG blocking activity for each of 6 different secreted protein assays (3 different Type I IFNs, IL-6, GM-CSF, and IFNλ, Fig. 3 and Table 1). Over a dozen blocking ACA have been identified in immunodeficiency disorders^14,21^, SLE^22,23^, COVID-19^8,11^, atypical infections^12^, and a variety of other disorders^24^. Even if many of the ACA are found not to have *in vitro* blocking activity, their high prevalence in COVID-19 patients and cohorts of patients with diverse infections suggests they may still play a role in disease pathogenesis (Fig. 1b). One possibility is that early in acute infection, levels of non-blocking ACA increase by the activation of pre-existing autoreactive B cells, driven by local production of the targeted cytokine which serves as an autoantigen. The resulting immune complex indirectly inhibits cytokine binding to its receptor by decreasing the local concentration of cytokines, reducing downstream signaling and enhancing pathogen replication and/or inflammation. If correct, this model has important therapeutic implications. For example, patients with anti-IFNα antibodies may benefit from treatment with exogenous IFNβ, which also binds and activates IFNAR and has not been identified as a prominent autoantigen in this study or in COVID-19^10^.

One of our most striking findings is the discovery that some patients acutely infected with influenza develop newly-detectable autoantibodies, an observation we recently described in hospitalized patients with COVID-19^10^. Autoantibodies including anti-SRP-54 and anti-thyroperoxidase (TPO) developed independently in 2 influenza patients and remained elevated approximately 1 month later, suggesting that viral infection directly triggered autoantibody development. Anti-SRP54 is associated with immune-mediated necrotizing myopathy (IMNM) and is a recognized biomarker for myositis. Multiple reports have linked prior infection to the onset of anti-SRP myositis. One study found that of 9 children with anti-SRP associated myositis, half were reported to have had an infection months before myositis onset^25^. Another case report described a 60-year-old woman diagnosed with influenza A infection a week before the onset of anti-SRP-associated IMNM^26^. The role of anti-SRP54 in myositis and how anti-SRP54 may be induced during infection, however, remains unknown. A comparison between the linear amino acid sequences for human SRP54 and either influenza or SARS-CoV-2 proteins identified no obvious homology. Interestingly, one study found that the SARS-CoV-2 nonstructural protein NSP8 interacts with the 7S RNA (7S ribonucleic acid) within the SRP complex, potentially displacing SRP54^27^. This interaction was found to disrupt proper trafficking of secreted and integral membrane proteins, contributing to significantly reduced IFN secretion.

Many other infectious agents have been linked epidemiologically and molecularly to the subsequent development of autoimmunity, including pandemic influenza^28^, Epstein Barr Virus (SLE^29^ and multiple sclerosis^30^), and dengue virus (antiplatelet antibodies and thrombocytopenia)^31^. When considering these well-described examples with published studies on COVID-19, and our current report on patients with a wide range of other infections, it appears that the potential infectious agents have for triggering specific autoantibodies may be much higher than previously recognized.

The mechanism(s) by which tolerance to self-antigens is broken, even if transiently, in COVID-19, influenza, and other infections is largely unknown. Molecular mimicry has been widely proposed in COVID-19 studies, with over 100 PubMed citations to date citing this mechanism; however, no convincing studies have yet to demonstrate this experimentally. Many ACA are detectable at the time of infection, and their levels appear to remain mostly constant (e.g., anti-IFNγ and anti-IFNα2) or increase modestly (e.g., anti-IFNα7, Fig. 4a) over time. Thus, molecular mimicry is unlikely to explain the large increases in levels of newly-detected ACA previously described in severely-ill COVID-19 patients, such as inducible ACA recognizing IL-22, IL-17, and IFNε^10^. Molecular mimicry is also unlikely to explain the development of anti-SRP-54 and anti-TPO in influenza and SARS-CoV-2 infection (Fig. 4b), as this mechanism would implicate that self-proteins cross-react with proteins from two unrelated respiratory viruses. Finally, autoimmune thyroiditis and anti-TPO are commonly observed following transplantation, cancer treatment with checkpoint inhibitors, and in many autoimmune diseases, arguing against an infection-specific mechanism.

A more likely explanation is that cytokines and interferons secreted in response to viral infection drive ACA production by pre-existing autoreactive B cells. We previously reported that APS-1 patients with serum anti-Type I IFN and IL-17A display in their blood an accumulation of autoreactive mature naïve B cells, some with measurable reactivity to Type I IFN and IL-17A^21^. Hence, early impairments of naïve B cell selection, associated with many autoimmune patients, may contribute to the production of ACA-expressing B cells and secretion of ACA, a response that may be enhanced during infection^32^. It remains to be determined whether potential defects in early B cell tolerance checkpoints in ICU patients with ACA result from genetic alterations, such as AIRE deficiency in APS-1 patients, promoting sustained serum ACA over time, or if these B cell tolerance defects are only transiently induced during infection.

Finally, autoantibodies have been postulated to play a role in a subset of COVID-19 survivors with “long haul” symptoms (termed post-acute sequela of COVID-19, or PASC) that have been well-described and are under active study^7,33^. Many PASC characteristics mirror the known long-term effects of sepsis and critical illness (often termed Post-ICU syndrome, or PICS), with a substantial proportion of patients still reporting diminished quality of life or new-onset neurologic and psychiatric deficits six and twelve months after acute illness^34^. Survivors of ARDS similarly report diminished functional status even one and five years after discharge, despite lung function returning to near-normal levels^35^. Data on the duration and clinical implications of autoantibodies in longer-term recovery of patients with PASC, PICS, and now ARDS, however, are lacking, and whether these lingering symptoms reflect persistent autoimmunity or inflammation is also unknown.

A key advance presented here is the widespread nature of autoantibodies that are seen across not only multiple respiratory viral infections but also non-respiratory bacterial infections observed in patients admitted to the ICU. A strength of our study is the availability of rich clinical data on all cohorts, enabling correlations between clinical data and autoantibody profiles. Patients in the Stanford ICU cohort were phenotyped by 3-physician review of each case to assign the most probable infection status (uninfected, viral, bacterial, fungal, or combination infections)^36^. It is important to note that clinical phenotyping for infection is known to be imprecise, with a cultured organism present only ~40–60% of the time^37,38^. In the current study, ~25% of the ICU population was clinically phenotyped as non-infected by 3-physician review. Some of these patients were admitted with syndromes such as chronic obstructive pulmonary disease (COPD) or asthma flares, and it is certainly possible that viral infections or bacterial superinfections preceding admission were not identified. Cohorts from Marburg, Giessen, and Athens were also deeply phenotyped, including clinical and laboratory testing, the identification of specific pathogen(s), clinical outcomes, and vaccination status.

Our study has several limitations that require future experiments. The number of subjects characterized is relatively small, and some patients who were described as “non-infected” may have been infected and not have displayed clinical signs of infection. Moreover, only one cohort of influenza-infected patients was available for longitudinal analysis. It is unknown whether newly-detectable autoantibodies are transient or permanent components of the new antibody repertoire, nor is it known if a subset of patients will go on to develop clinical manifestations such as myositis (e.g., anti-SRP54) or thyroiditis (anti-TPO) that are associated with the newly-detected autoantibody specificity. The acute impact of these autoantibodies on ICU presentation is not clear in these cohorts, as patients with autoantibodies were not obviously more ill (e.g., rates of shock and mortality were similar), although most patients in these cohorts had severe disease. Future cohorts that include outpatients with mild disease will help inform the incidence across the spectrum of illness severity^34,35^. Except for one influenza-infected patient with blocking interferon ACA who later developed severe COVID-19, pre-infection samples were unavailable in our cohorts to definitively determine whether autoantibodies pre-dated infection. Once SARS-CoV-2 is no longer the dominant circulating pathogen, future studies of influenza and other seasonal infections will be necessary to accurately measure the prevalence of ACAs and newly-detectable autoantibodies against secreted proteins and CTD antigens. Finally, we do not have paired PBMCs to correlate our autoantibody findings with analyses of specific immune cell populations, particularly to explore defects in autoreactive B cell subsets.

In summary, the scale of the COVID-19 pandemic and the availability of well-annotated, longitudinally-collected biospecimens has enabled unprecedented ability to analyze how a specific pathogen causes severe disease and death in some patients versus asymptomatic infection in the majority of others. The studies described in this report significantly extend discoveries on COVID-19 that have advanced our understanding of autoimmunity and autoantibodies. Future experiments are needed to determine whether our results extend into an ambulatory setting, and if so, whether the immunologic mechanisms related to the breaking of tolerance to self-molecules are shared with genetic disorders, such as APS-1, and/or inducible autoimmunity, as observed in the patients treated with checkpoint inhibitors who experience adverse events. Finally, early identification of ICU patients with pathogenic autoantibodies and infections may alter not only how we think about ICU infection – as an autoimmune disorder in some patients – but also how the underlying autoimmune disorder is treated in the setting of infection.

## Methods

### Serum and plasma samples

Serum or plasma samples were obtained following informed consent. Samples were cryopreserved at −80°C until antibody profiling was performed. Clinical and laboratory characteristics of the cohorts can be found in Supplementary Tables 2–6.

#### Stanford ICU patients.

The Stanford ICU biobank is a collection of whole blood samples prospectively obtained from subjects admitted to Stanford Hospital ICU with at least one risk factor for ARDS (e.g., sepsis, aspiration, and/or trauma). Exclusion criteria included routine post-operative patients and severe anemia. Clinical data were abstracted from the medical record by study staff blinded to autoantibody levels. Infection status was determined through retrospective chart review by three physicians blinded to autoantibody levels. Positive or negative infection status was defined by at least 2/3 physician consensus as previously described^36^. Samples used in this study were collected between February 2015 and November 2018, and all patient samples and data collected were compliant with the Stanford Institutional Review Board (n = 167, Stanford IRB #28205). See Supplementary Table 2 for details.

#### Giessen and Marburg patients with acute respiratory illnesses.

Serum samples were obtained from hospitalized subjects at 2 academic centers in Germany (Giessen and Marburg). Serum samples from patients admitted to the ICU between April and May 2020 for COVID-19 symptoms but who tested negative by PCR (n = 19) and serum samples from patients hospitalized with Influenza A and pneumonia between January 2018 and March 2020 (n = 25) were obtained from the Philipps University Marburg (IRB# 57/20). Serum samples from ARDS patients (n = 17) were obtained from the University of Giessen and were collected between July 2016 and January 2020 (IRB# 58/15) See Supplementary Tables 4–6 for details. Using a viral array recently described by our group^10^, we identified one patient who tested negative for SARS-CoV-2 based on PCR but was positive for antibodies against SARS-CoV-2 spike protein (Supplementary Fig. 6). This patient was excluded from subsequent analyses.

#### Acute influenza infection.

Patients were admitted to the Sotiria Thoracic Diseases Hospital of Athens (approval numbers 16707/10-7-18) and the Attikon University Hospital of the University of Athens Medical School (approval number 1821A/22-9-16) in Greece between December 2018 and April 2019 and were diagnosed with acute influenza by the BioFire FilmArray Respiratory Panel test (bioMerieux, BIOFIRE FilmArray Respiratory Panel (RP) Cat#: RFIT-ASY-0124) (n = 40). Serum samples were collected from each patient at one to three timepoints. Samples from the first timepoint (T1) were collected on the day that the patient was admitted to the hospital and diagnosed with influenza. Samples from the second timepoint (T2) were collected approximately a week later. Samples from the third timepoint (T3) were collected approximately a month after hospital admission. Serum was collected at T1 for all 40 patients, while serum was collected at T2 and T3 for 29 and 20 patients, respectively. See Supplementary Table 3 for details.

#### COVID-19 samples.

Serum samples which had been characterized previously using protein arrays were obtained from hospitalized patients with COVID-19 from Philipps University Marburg between April and June 2020 (n = 18, IRB#57/20)^10^. Two samples with high levels of ACA were selected to develop blocking assays.

#### Healthy controls.

Serum and plasma samples from anonymous healthy controls (HC, n = 33) were obtained prior to the COVID-19 pandemic from Stanford Blood Bank and Stanford Hospital and Clinics. Normal human sera (ImmunoVision, Product # HNP-0300, certified to be nonreactive to Hep-2 cell lysates at a titer of 1:100), was used for validation and as negative controls in array experiments.

#### Positive control subjects with known autoimmune disease and known blocking autoantibodies.

Prototype human plasma samples derived from participants with autoimmune diseases with known reactivity patterns (e.g., ds-DNA, Scl-70, centromere, SSA, SSB, cardiolipin, whole histones, and RNP) were purchased from ImmunoVision or were obtained from Stanford Autoimmune Diseases Biobank and Oklahoma Medical Research Foundation (a gift of Dr. Judith James). Serum from patients with autoimmune polyglandular syndrome type 1 (APS-1), IPEX, pulmonary alveolar proteinosis, and atypical mycobacterial infections^14^ were provided by Dr. David Lewis (Stanford) and used for array experiments and for blocking studies.

### Bead-based antigen arrays

Two different custom bead-based antigen arrays were created, as previously described^21,22,39–44^. A complete list of all antigens, vendors, and catalogue numbers can be found in Supplementary Tables 1 ***and*** 7. “Traditional Autoantigen Arrays” included 55 commercial protein antigens associated with connective tissue diseases (CTDs) (Supplementary Table 7). “Cytokine Arrays” comprised 58 proteins, including cytokines, chemokines, growth factors, acute phase proteins and cell surface proteins (Supplementary Table 1). Arrays were constructed as previously described. Briefly, antigens were coupled to carboxylated magnetic beads (MagPlex-C, Luminex Corp.), each with unique barcodes^39,40^. Immobilization of some antigens and control antibodies on the correct bead IDs was confirmed using commercially available mouse monoclonal antibodies or antibodies specific for engineered epitope tags. Prototype human plasma samples were used for validation of bead arrays.

#### Array probing

Serum or plasma samples were tested at 1:100 dilution in 0.05% PBS-Tween supplemented with 1% (w/v) bovine serum albumin (BSA) and transferred into 96-well plates in a randomized layout. The bead array was distributed into a 384-well plate (Greiner BioOne) by transfer of 5 μl bead array per well. 45 μl of the 1:100 diluted sera were transferred into the 384-well plate containing the bead array. Samples were incubated for 60 min on a shaker at room temperature. Beads were washed with 3 × 60 μl PBS-Tween on a plate washer (EL406, Biotek), and 50 μl of 1:500 diluted R-phycoerythrin (R-PE) conjugated Fc-γ-specific goat anti-human IgG F(ab’)2 fragment (Jackson ImmunoResearch) was added to the 384-well plate for detection of bound human IgG. After incubation with the secondary antibody for 30 min, the plate was washed with 3 × 60 μl PBS-Tween and re-suspended in 50 μl PBS-Tween prior to analysis using a FlexMap3D^™^ instrument (Luminex Corp.). Binding events were displayed as Mean Fluorescence Intensity (MFI). All samples were run in duplicate in each experiment. Longitudinal samples which showed new onset autoantibodies were reanalyzed in duplicate on new bead arrays to confirm results. Samples from patients with COVID-19 were heat-inactivated prior to analysis, as previously described^45^.

#### Cytokine Blocking Assays

##### pSTAT induction in cell-based assay.

The blocking activity of patient sera with specific anti-cytokine autoantibodies was assessed as previously described^8^. Cells (400,000 cells/condition) were incubated with 10% healthy control serum, patient serum or plasma, commercial blocking antibody, positive control blocking serum, or media only for 15 minutes and stimulated with the appropriate cytokine. The percentage of pSTAT-positive cells was compared between the stimulated and unstimulated condition. To develop each assay, cells were stimulated at different cytokine concentrations to determine the final working concentration. The lowest concentration at which maximal stimulation was observed was selected for the final blocking assays (Supplementary Fig. 5a). Patient sera positive by array for anti-IFNα2, -IFNα7, -IFNα8, -IFNγ, -IL-6, and -GM-CSF antibodies were assessed using blocking assay conditions summarized in Supplementary Table 8. Cells were assessed on a BD LSR II analyzer and analyzed using FlowJo software version 10.8. A complete list of cytokines, blocking antibodies, staining antibodies, vendors, and catalogue numbers can be found in Supplementary Table 9.

##### GFP reporter assays.

The activities of IFNα2, IFNγ, IFNλ2, and IFNλ3 were detected by a HAP1 reporter cell line^46^. This cell line expresses GFP under the control of the ISRE (interferon-stimulated response element) of the IFIT2 gene (interferon-induced protein with tetratricopeptide repeats 2) and is sensitized for IFNλ detection by stable overexpression of IFNLR1^46^. HAP1 reporter cells were cultured with complete IMDM media containing 10% FBS and 1% penicillin/streptomycin/amphotericin. HAP1 reporter cells were seeded into 48-well plates with 3×10^4^ cells per well and incubated overnight. To evaluate the function of HAP1 reporter cells of indicating the activities of IFNs, IFNs were prepared into a 5-fold serial dilution in serum-free IMDM media and added to the HAP1 reporter cells (Supplementary Fig. 7a). The cells were incubated 22–24 hours at 37°C with 5% CO_2_, treated with 0.25% trypsin, suspended into single cells, and analyzed by flow cytometry (Cytek Aurora) for the expression of GFP. To evaluate the neutralization activity of monoclonal antibodies to each IFN, the antibodies were prepared by a 2-fold serial dilution in serum-free IMDM (Supplementary Fig. 7, b ***and*** c). To evaluate the neutralizing activity in the serum samples, serum was heat-inactivated at 56°C for 30 minutes and prepared into a 5-fold serial dilution. The serial-diluted monoclonal antibodies and serum were cultured with their cognate interferons for 1 hour at room temperature. The concentrations of IFNα2, IFNγ, IFNλ2, and IFNλ3 during the incubation were 80 U/ml, 16 U/ml, 2 ng/ml, and 2 ng/ml, respectively. The IFN-antibody and IFN-serum mixtures were then added into HAP1 reporter cells, and the final concentrations of IFNα2, IFNγ, IFNλ2, and IFNλ3 in the cell culture were 40 U/ml, 8 U/ml, 1 ng/ml, and 1 ng/ml, respectively. GFP expression in the cells was evaluated 22–24 hours after incubation as described above. A complete list of the cytokines and monoclonal antibodies used, and their vendors and catalogue numbers, can be found in Supplementary Table 10.

#### Statistical Analyses

R, RStudio, and various R packages were used to perform analyses^47,48^. For normalization, MFI values for “bare bead” IDs were subtracted from MFI values for antigen conjugated bead IDs, and replicate MFI values were averaged. The average MFI for each antigen was calculated using samples from healthy subjects (all obtained before December 2019). Serum samples were considered “positive” for antibodies recognizing a specific antigen if the normalized MFI was > 5 standard deviations (SD) above the average MFI for HC for that antigen, and if the normalized MFI was >3,000 units, a more stringent threshold than those commonly published in related literature^8^. Based on the Significance Analysis of Microarrays (SAM) algorithm^49^, statistically significant antigens were identified using false discovery rate (FDR)-adjusted p-values (q < 0.001), 2-fold change cutoffs, and 10,000 permutations. Data were visualized in GraphPad Prism v.9.3.0 (345). Complexheatmap v.2.8.0 was used for all heatmaps^50^. Upon publication of this study in a peer-reviewed journal, de-identified array data will be uploaded to the Gene Expression Omnibus (GEO) database.

Within the Stanford ICU cohort, analyses evaluated predictors for the development of autoantibodies as well as pertinent ICU outcomes related to the presence of autoantibodies in patient serum. Fisher’s Exact Test was used to evaluate the association between age (<60 vs ≥60), sex, immunocompromised status, white blood cell count (<12 vs ≥12K/uL), and infection status with the development of ACA and CTD-AAb. To control for interactions between variables, backwards stepwise logistic regression was performed, evaluating sex, age, infection status, and APACHE II score as predictors for the presence or absence of ACA and CTD-AAb. Fisher’s Exact Test was used to evaluate the association between autoantibodies and the development of shock, intubation, and 30-day mortality.

For the GFP reporter blocking assay, the activities of IFNα2, IFNγ, IFNλ2 and IFNλ3 were evaluated by measuring the percentages of GFP^+^ HAP1 reporter cells. The reduction of GFP signal due to the blocking activity of monoclonal antibodies or patient serum was calculated by subtracting the background signal (the percentage of GFP^+^ cells in the cell culture without any treatment) and then dividing by the maximal cytokine-induced signal (the percentage of GFP^+^ cells when cells were cultured with IFN alone). The reduction of GFP signal for each condition was plotted and four-parameter inhibitory dose-response curves were fitted to the data using GraphPad Prism v.9.3.0 (345). The half-maximal inhibitory concentration (IC50) was calculated using the equation,

(1) $$f\left(x\right)=Min+\frac{Max-Min}{1+\left(\frac{IC50}{x}\right)\text{Hill}},$$

where f(x) is the reduction of GFP signal, x is the concentration of the antibody in pg/ml, “Min” and “Max” are the plateau values of the Y axis, “Hill” is the Hill coefficient, and IC50 is the concentration of antibody where the reduction of GFP signal is halfway between the “Min” and “Max” values.

## Supplementary Material

Supplement 1

## Figures and Tables

**Table 1.** Clinical Characteristics of Patients with Detected Blocking Anti-Cytokine Antibodies.

Patient ID	Infection	Blocking ACA	Binding ACA	Age	Sex	Comorbidities	Status	Other
UMR15	Influenza	IFNα2	IFNα2, IFNα8, IFNα10	66	M	COPD, arterial hypertension, chronic heart disease	Alive	Two years after developing influenza, developed severe COVID-19 ARDS two weeks after the first Biontech mRNA vaccination, with re-activation of CMV and HSV-pneumonitis Slightly elevated CRP, normal total leukocytes, lymphopenia Vaccinated for flu in 2Ol8
UMR12	Influenza	GM-CSF	IFNα7, GM-CSF, sRANK-ligand	86	F	No significant medical history	Alive	Elevated CRP, leukopenia, normal lymphocyte percentage Not vaccinated for flu
AA9	Influenza A H3	IFNα2	IFNα1, IFNα2, IFNα7, IFNα8, IFNα10, IFNω, IL-11	73	M	COPD, sleep-breathing disorder, cardiovascular disease, heart failure, hypertension, dyslipidemia	Discharged	Ex-smoker Fever, rigor productive cough, bilateral lung infiltrates Not vaccinated for flu
AA20	Influenza A H3	IL-6	IL-6	77	F	Obesity, hypertension, atrial fibrillation, COPD	Discharged	Smoker Fever, productive cough, bilateral lung infiltrates Vaccinated for flu
SU107	Serratia bacteremia	IFNα2	IFNα1, IFNα2, IFNα7, IFNα8, IFNα10, IFNλ3, IFNω, IL-12p70	69	M	N/A	Alive	Intubated
SU019	Clinically suspected bacterial infection	GM-CSF	IFNα6, IFNα6, GM-CSF	70	F	N/A	Alive	Back/costovertebral angle tenderness, sepsis, dysuria Not intubated
SU047	MSSA pneumonia	IFNλ2, IFNλ3	IFNλ2, IFNλ3	69	M	Recurrent cholangitis	Alive	Following insulinoma resection, developed leukocytosis and worsening respiratory distress, requiring intubation. Bronchoscopy showed MSSA Pneumonia. Survived after l month in the hospital, but required several readmissions for recurrent VRE retroperitoneal abscesses (now resolved)
SU008	RSV	IFNα7	IFNα7, IFNα8, IFNα10	63	F	N/A	Alive	Fever, leukocytosis, acute respiratory failure Not intubated
SU042	None reported	IL-6	IFNα7, IL-4, IL-6, IL-17F, MIP-1α	84	F	N/A	Alive	Not intubated
SU080	Clostridioides difficile	GM-CSF	IFNα10, GM-CSF	60	F	N/A	Alive	Shock Not intubated
UGMLC5	Suspected bacterial infection, pathogen not detected	IFNα8	IFNα7, IFNα8, IFNα10	69	M	N/A	N/A	Pneumonia-induced ARDS

## Data Availability

All raw and normalized array data will be made available upon publication in a peer-reviewed journal by depositing in the GEO database. Clinical data other than data already shown in Supplementary Tables 2–6 are not available, to remain compliant with HIPAA requirements.
