# Supplementary material for: Autoantibodies targeting cytokines and connective tissue disease autoantigens are common in acute non-SARS-CoV-2 infections
Source: Res Sq. 2022 Jan 20:rs.3.rs-1233038. Preprint. [Version 1] doi: 10.21203/rs.3.rs-1233038/v1 (PMC8786233; doi:10.21203/rs.3.rs-1233038/v1)
Supplement: Supplement 1 [file 68b6d00cbd7b8d4d71e1bdfa.docx]

**SUPPLEMENTARY FIGURES**





**Supplementary Fig. 1: High prevalence of ACA in hospitalized ICU patients compared to HC regardless of infection status.** Tukey box plots comparing MFI data from Stanford ICU patients (n = 167) and HC (n = 22) for nine antigens for which statistically significant differences were determined between ICU patients (regardless of presence or absence of infection) and HC using two-tailed Wilcoxon rank-sum tests with Bonferroni correction. The middle line represents the median, while the lower and upper hinges correspond to the first and third quartiles. The upper whisker extends from the hinge to 1.5 times the interquartile range (IQR) above the 75th percentile MFI value, and the lower whisker extends from the hinge to 1.5 times the IQR below the 25th percentile MFI value.


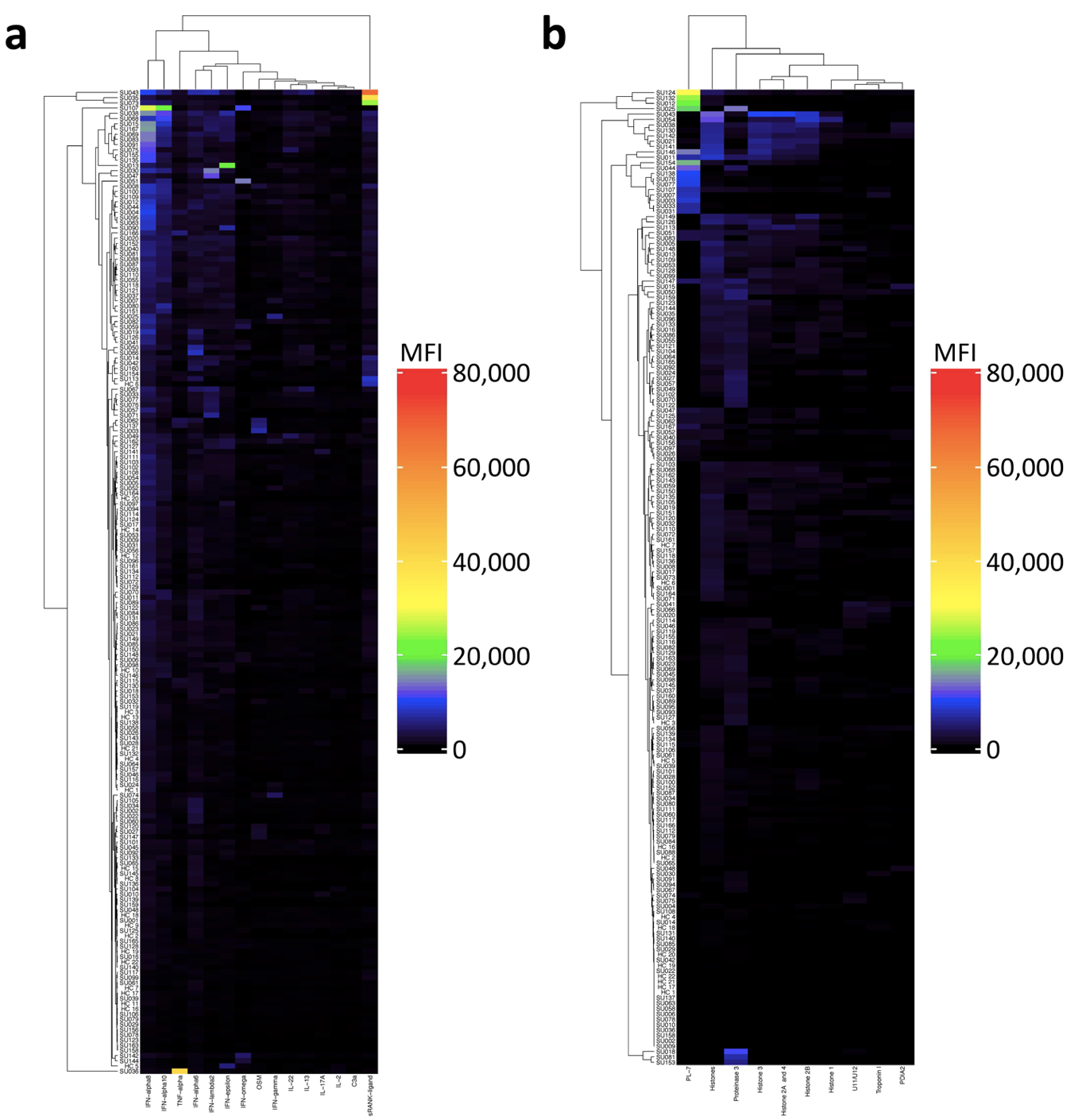

**Supplementary Fig. 2: Array analysis of Stanford ICU patients using the SAM algorithm.** Clustered heatmap representing antigens with statistically significantly higher reactivity in Stanford ICU patient subjects compared to HC. Statistically significant antigens were identified using false discovery rate (FDR)-adjusted p-values (q < 0.001), 2-fold change cutoffs, and 10,000 permutations in the SAM algorithm. **a** 15 of 58 antigens in the cytokine array were identified with higher reactivity in ICU patients (n = 167) compared to HC (n = 22). **b** 10 of 55 antigens in the traditional autoantigen array had higher reactivity in ICU patients (n = 167) compared to HC (n = 14).


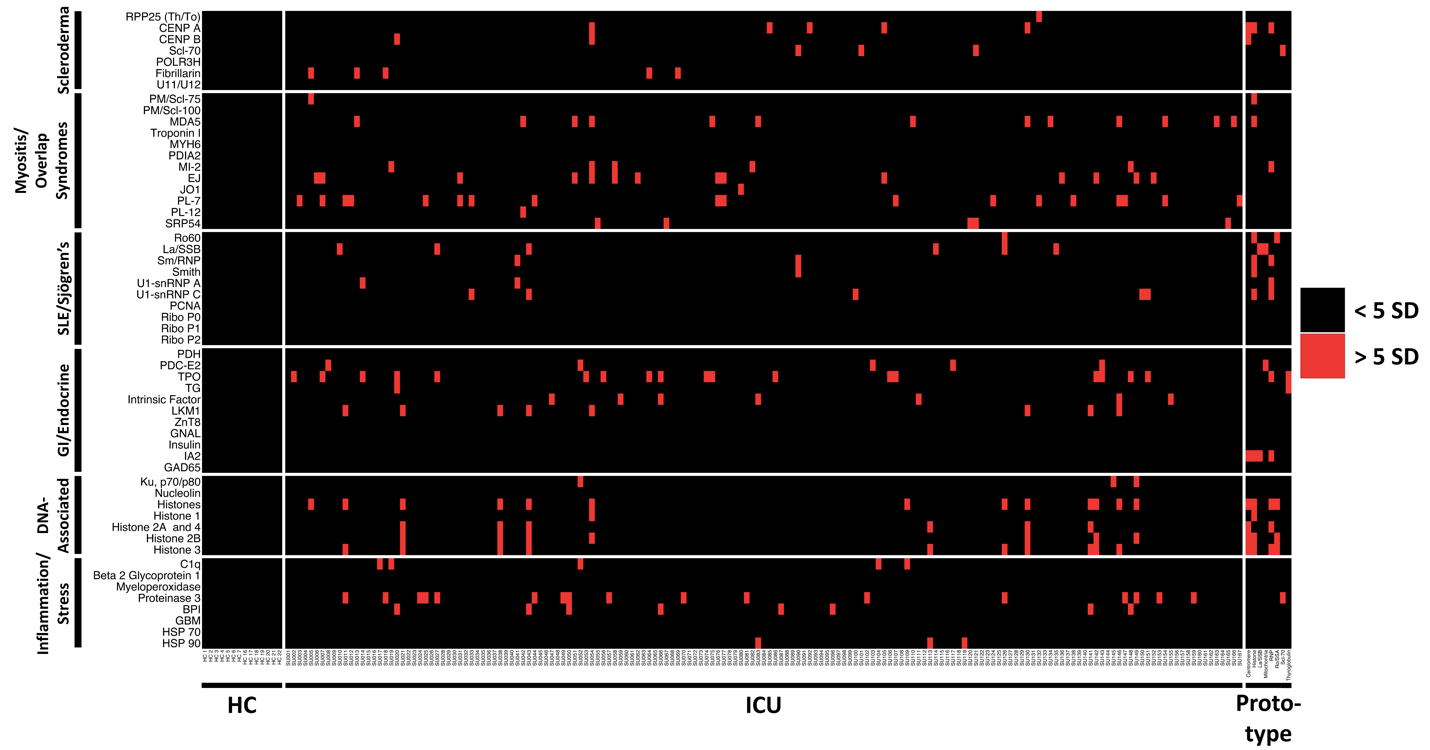
**Supplementary Fig. 3: Antibodies targeting traditional connective tissue disease (CTD) autoantigens in hospitalized ICU patients.** Heatmap representing serum IgG antibodies measured in Stanford ICU patient subjects (n = 167), HC (n = 22), and patients with prototype autoimmune disorders (n = 8) using a 55-plex autoantigen array. Antigens are grouped on the y-axis by disease category (scleroderma, myositis and overlap syndromes such as mixed connective tissue disease (MCTD), SLE and Sjögren’s, gastrointestinal and endocrine disorders); association with chromatin; and association with tissue inflammation or stress responses. Colors indicate autoantibodies whose MFI measurements are > 5 SD (red) or < 5 SD (black) above the average MFI for HC. MFIs <3,000 were excluded.


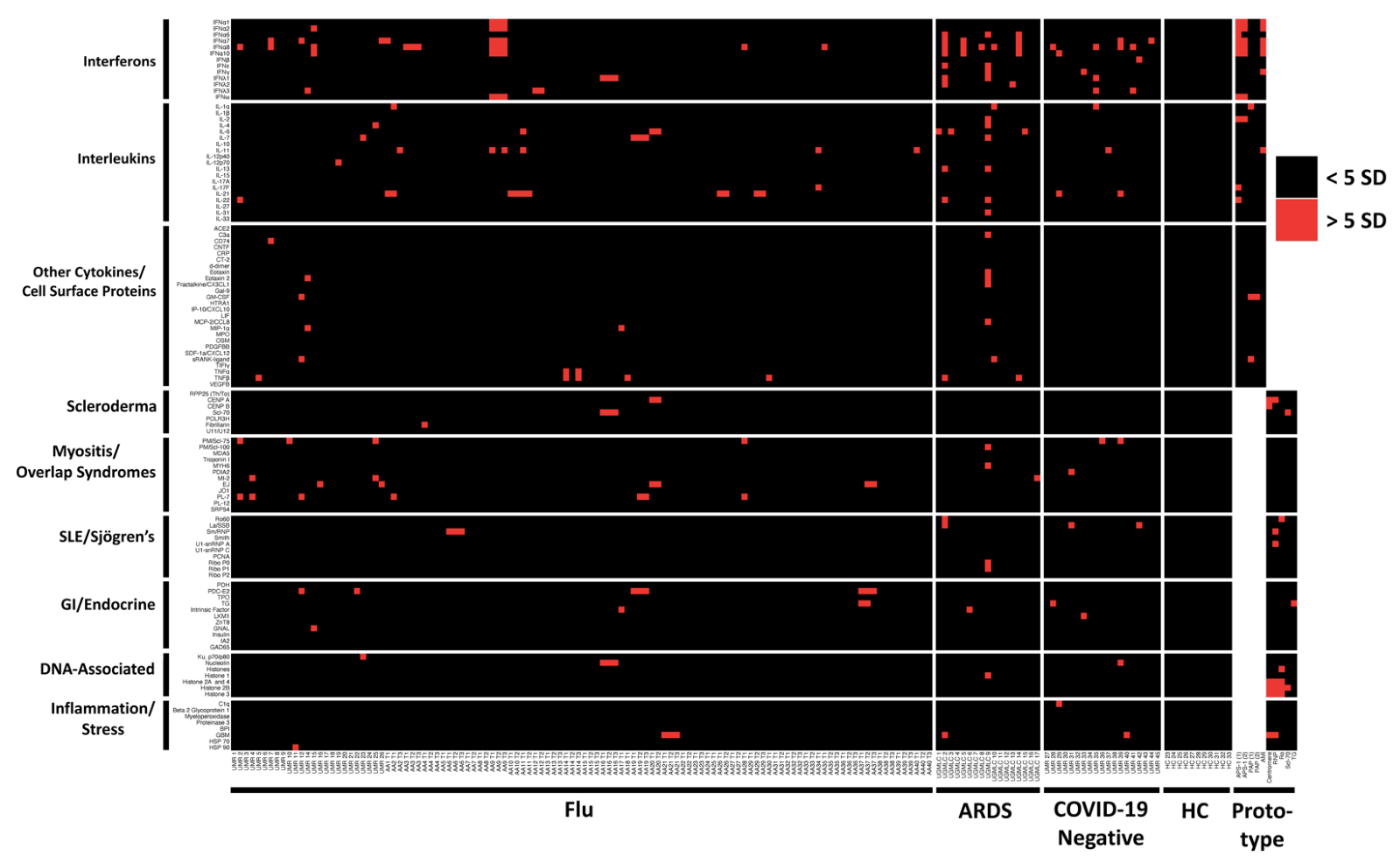
**Supplementary Fig. 4: Autoantibodies in ICU patients and patients with influenza.** Heatmap representing serum IgG antibodies measured in influenza patients (n = 25); ARDS patients (n = 17), both collected prior to the COVID-19 pandemic; ARDS patients who tested negative for COVID-19 (n = 19); and HC (n = 11) using a 58-plex cytokine array (upper panels) and 55-plex traditional CTD autoantigen array (lower panels). Prototype serum samples (n = 5 on both arrays) were also tested. Cytokines are grouped on the y-axis by category (interferons, interleukins, and other cytokines/growth factors/receptors). Connective tissue disease antigens are grouped on the y-axis by disease category (scleroderma, myositis and overlap syndromes such as mixed connective tissue disease (MCTD), SLE and Sjögren’s, gastrointestinal and endocrine disorders); association with chromatin; and association with tissue inflammation or stress responses. Colors indicate autoantibodies whose MFI measurements are >5 SD (red) or <5 SD (black) above the average MFI for HC. MFIs <3,000 were excluded.


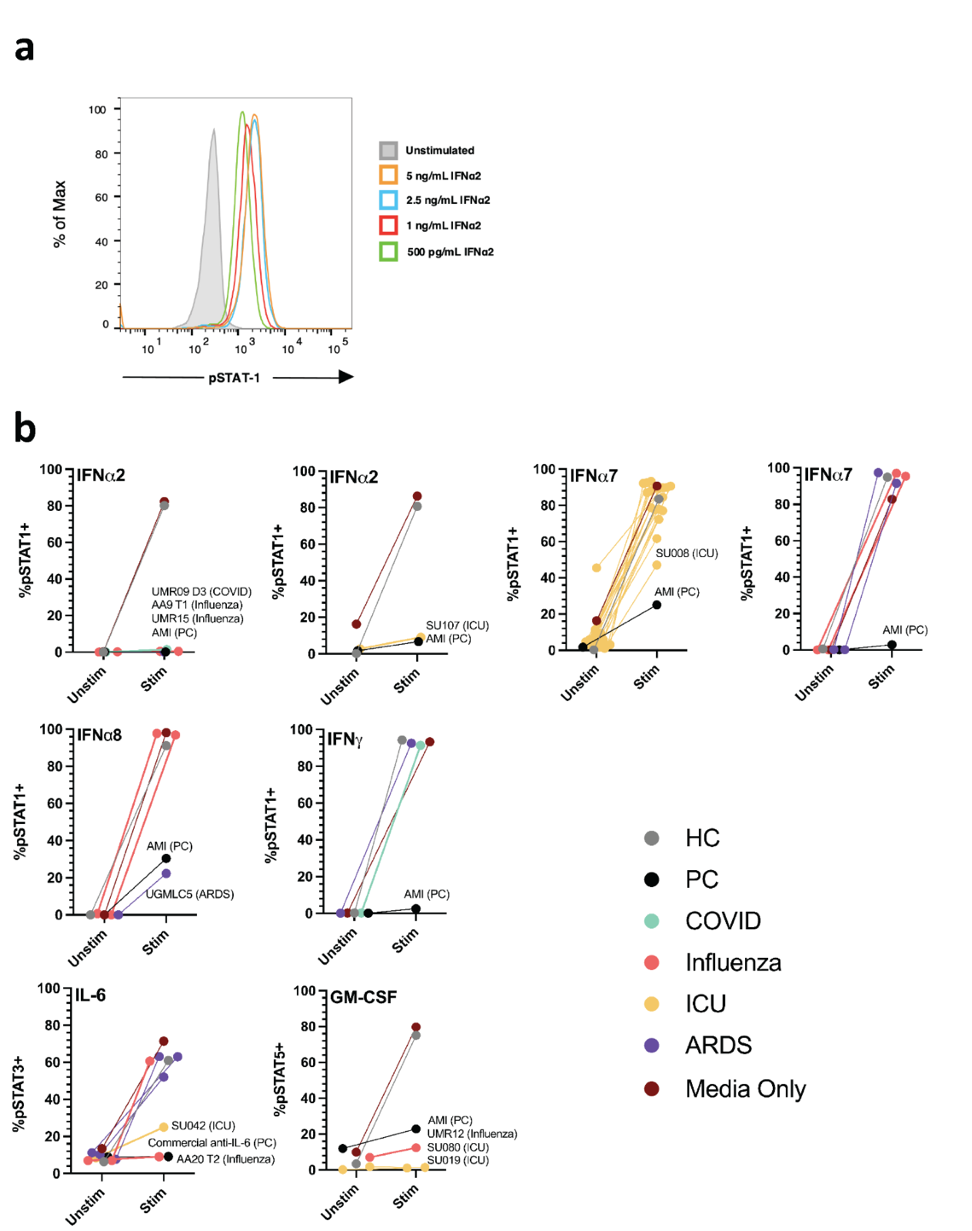


**Supplementary Fig. 5: Blocking Assay Development and Results. a**Representative fluorescence-activated cell sorting (FACS) plots of IFNα2 blocking assay. U937 cells were treated with various concentrations of recombinant IFNα2 to identify a concentration (2.5 ng/mL) that would yield maximal stimulation in the final assay (***Supplementary Table 8***). **b**Blocking activity of patient serum on cells in cytokine signaling assays, reported as percentage of pSTAT positive cells in the unstimulated and stimulated condition. Patient sera were from COVID-19 (n=2), influenza (n_Giessen/Marburg_ = 4, n_Athens_ = 5), Stanford ICU (n_infected_ = 19, n_non-infected_ = 2) and ARDS (n = 8) patients. Healthy controls (HC) and positive controls (PC: commercially available blocking antibody or prototype serum from a patient with atypical mycobacterial infection [AMI]) are also included. Independent experiments are shown. Samples with complete or partial blocking activity are labeled.


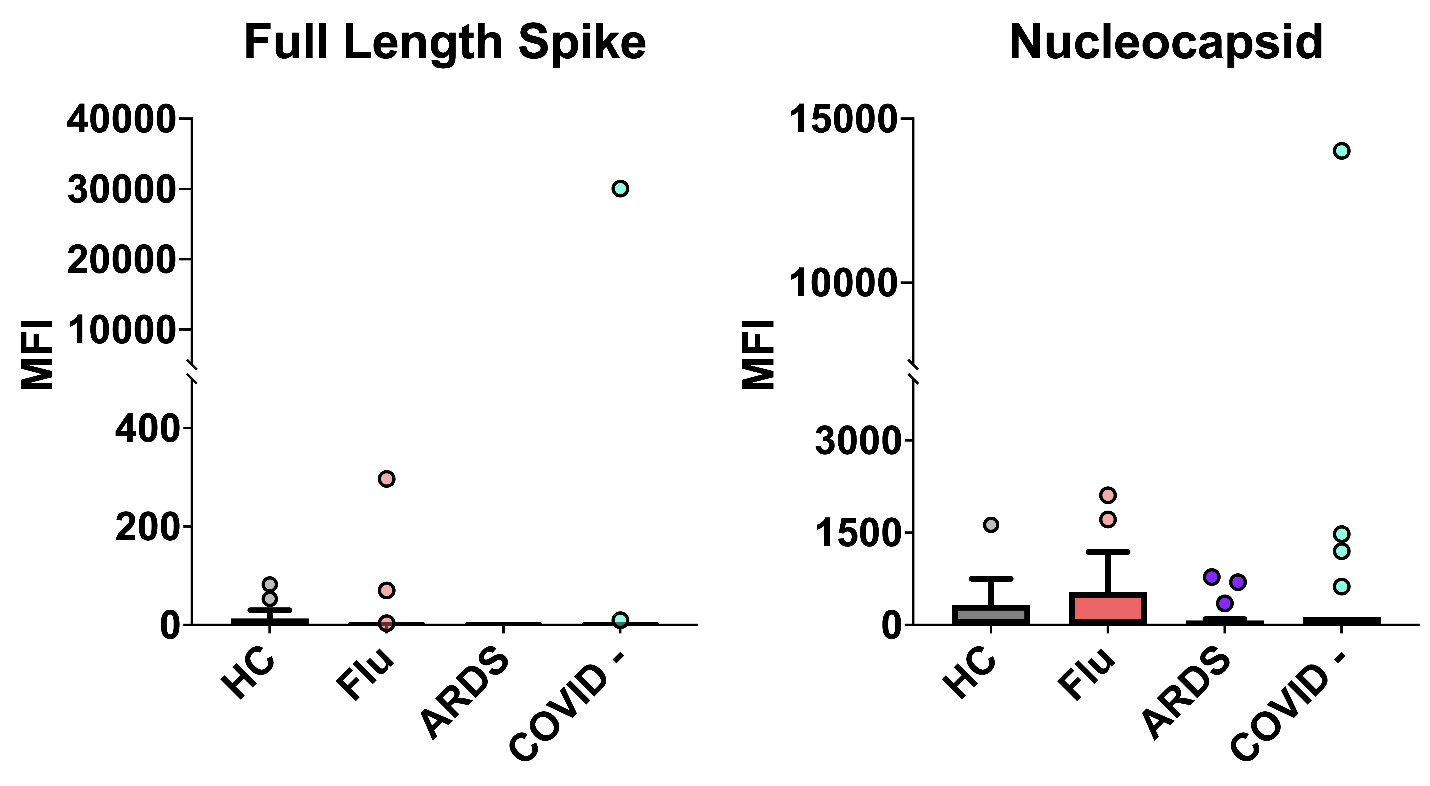

**Supplementary Fig. 6: A COVID-19 PCR-negative patient from the Marburg cohort has high levels of antibodies targeting SARS-CoV-2 proteins**. Tukey box plots comparing MFI data for SARS-CoV-2 Spike and Nucleocapsid proteins from our viral array^10^ in influenza patients (n = 25) and ARDS patients (n = 17), both collected prior to the COVID-19 pandemic; ARDS patients who were COVID-19- (n = 19); and HC (n = 11).  One patient who tested negative for COVID-19 by PCR was found to have high levels of antibodies against SARS-CoV-2 spike and nucleocapsid proteins. The middle line represents the median, while the lower and upper hinges correspond to the first and third quartiles. The upper whisker extends from the hinge to 1.5 times the interquartile range (IQR) above the 75th percentile MFI value, and the lower whisker extends from the hinge to 1.5 times the IQR below the 25th percentile MFI value.


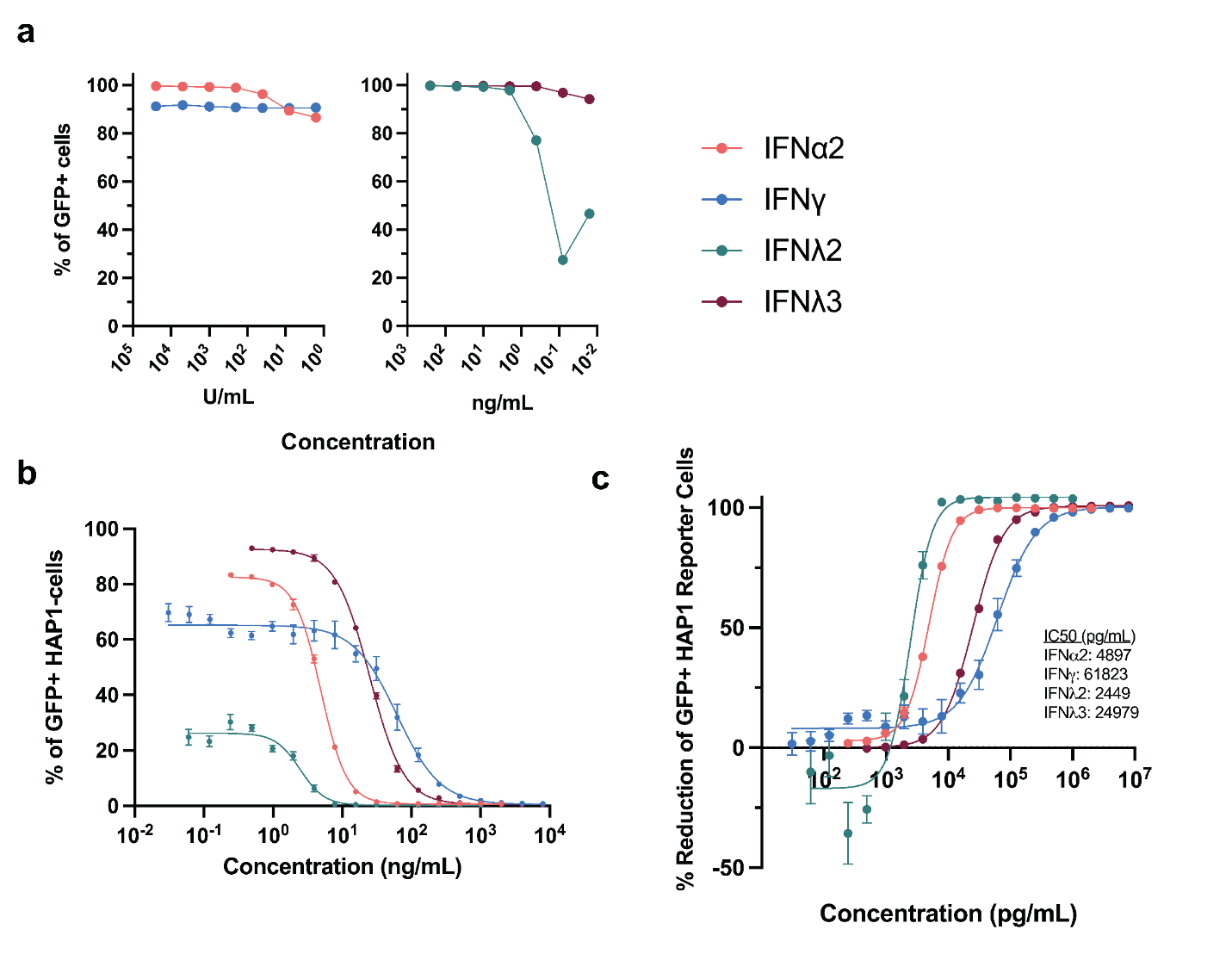
**Supplementary Fig. 7: HAP1 reporter cell line allows the detection of type I, II and III interferons.** **a** A human haploid reporter cell line that contains the ISRE from IFIT2 (HAP1 ISRE-GFP) was used to detect IFNs with flow cytometry 22-24 hours after incubation with IFNs. **a** Dose-dependent response to IFNα2, IFNγ, IFNλ2 and IFNλ3. **b and c** Neutralization of IFNα2 (40 U/ml), IFNγ (8 U/ml), IFNλ2 (1 ng/ml), and IFNλ3 (1 ng/ml) with cognate monoclonal antibodies. Triplicates for each condition. Reduction of the percentages of GFP+ cells as in (C) was calculated by subtracting the background (no cytokines) and dividing by the percentage of GFP+ cells when the cytokines are completely functional (not incubated with neutralizing antibodies). Calculation of IC50s was done by fitting four-parameter inhibitor dose-response curves to the data, as calculated above, using GraphPad Prism v.9.3.0 (345).

**SUPPLEMENTARY TABLES**

**Supplementary Table 1. Cytokine Array content.**

| **Bead ID** | **Antigen** | **Vendor** | **Catalog #** |
| --- | --- | --- | --- |
| 1 | Bare Bead |  |  |
| 2 | Human IgG from serum | Sigma | I4506 |
| 3 | Anti-Human IgG Fc fragment specific | Jackson | 109-005-008 |
| 4 | Anti-Human IgG (H+L) | Jackson | 109-005-003 |
| 5 | Anti-Human IgG F(ab') fragment specific | Jackson | 109-005-006 |
| 6 | CD74 | Prospec | PRO-1467 |
| 7 | IFNλ2 | Peprotech | 300-02K |
| 10 | IL-1α | Prospec | CYT-253 |
| 13 | IFNα1 | Prospec | CYT-291 |
| 14 | IFNα7 | Prospec | CYT-196 |
| 16 | IFNα10 | Sino Biological | 10349-H08H |
| 18 | IFNα8 | Sino Biological | 10347-H08H |
| 19 | IFNα6 | Origene | TP760329 |
| 20 | IL-2 | Peprotech | 200-02 |
| 21 | IL-4 | Peprotech | 200-04 |
| 22 | IL-13 | Peprotech | 200-13 |
| 23 | IL-21 | Peprotech | 200-21 |
| 24 | Fractalkine/CX3CL1 | Peprotech | 300-31 |
| 25 | IP-10/CXCL10 | Peprotech | 300-12 |
| 26 | IL-31 | Prospec | CYT-625 |
| 27 | IL-6 | Prospec | CYT-098 |
| 28 | MCP-2/CCL8 | Peprotech | 300-15 |
| 29 | OSM | Peprotech | 300-10 |
| 30 | IL-11 | Prospec | CYT-214 |
| 31 | SDF-1a/CXCL12 | Peprotech | 300-28A |
| 32 | IL-27 | Prospec | CYT-048 |
| 33 | CNTF | Prospec | CYT-272 |
| 34 | CT-2 | Prospec | PRO-1578 |
| 38 | GM-CSF | Peprotech | 300-03 |
| 39 | IFNα2 | R&D | 11101-2 |
| 40 | IFNβ | Peprotech | 300-02BC |
| 41 | IFNγ | Peprotech | 300-02 |
| 42 | IFNε | R&D | 9667-ME-025/CF |
| 43 | IFNλ1 | Peprotech | 300-02L |
| 44 | IFNλ3 | R&D | 5259-IL-025/CF |
| 45 | IFNω | R&D | 11395-1 |
| 46 | IL-10 | Peprotech | 200-10 |
| 47 | IL-12p40 | Peprotech | 200-12P40 |
| 48 | IL-12p70 | Peprotech | 200-12 |
| 49 | IL-15 | Peprotech | 200-15 |
| 50 | IL-17F | Peprotech | 200-25 |
| 51 | IL-1β | Peprotech | 200-01B |
| 52 | IL-22 | Peprotech | 200-22 |
| 55 | TNFα | Peprotech | 300-01A |
| 56 | TNFβ | Peprotech | 300-01B |
| 58 | ACE2 | Sino Biological | 10108-H05H |
| 59 | Eotaxin | Peprotech | 300-21 |
| 60 | Eotaxin 2 | Peprotech | 300-33 |
| 62 | IL-17A | Peprotech | 200-17 |
| 63 | IL-33 | Peprotech | 200-33 |
| 64 | IL-7 | Peprotech | 200-07 |
| 65 | MIP-1α | Peprotech | 300-08 |
| 67 | PDGFBB | Peprotech | 100-14B |
| 68 | sRANK-ligand | Peprotech | 310-01C |
| 69 | TIFIγ | Surmodics | A11001 |
| 70 | CRP | Prospec | PRO-335 |
| 71 | MPO | Prospec | ENZ-074 |
| 73 | C3a | R&D | 3677-C3-025 |
| 76 | Gal-9 | R&D | 9064-GA-050 |
| 77 | LIF | Peprotech | 300-05 |
| 78 | VEGFB | Peprotech | 100-20B |
| 79 | HTRA1 | R&D | 2916-SE-020 |
| 80 | d-dimer | LeeBio | 200-13-0.1 |

**Supplementary Table 2. Stanford ICU patient cohort clinical characteristics.**

|  | ICU Patients (n = 167) |
| --- | --- |
| Age [Median (IQR)] | 66 (54 – 75) |
| Sex [Percent; (N)] |  |
| Female | 42.5% (71) |
| Male | 57.5% (96) |
| Race/Ethnicity [Percent; (N)] |  |
| Asian/Pacific Islander | 10.2% (17) |
| Black/African American | 4.8% (8) |
| Hispanic | 19.8% (33) |
| White | 57.5% (96) |
| Other/Unknown | 7.7% (13) |
| Infection [Percent; (N)] | 69.5% (116) |
| Median SAPS 3 [Median (IQR)] | 67 (55 – 80) |
| Median APACHE II [Median (IQR)] | 24 (19 – 32) |
| Shock [Percent; (N)] | 58.7% (98) |
| 30-day Mortality [Percent; (N)] | 25.1% (42) |

**Supplementary Table 3. Athens influenza patient cohort clinical characteristics.**

|  | **Influenza Patients (N = 40)** |
| --- | --- |
| **Age [Median (IQR)]** | 49.5 (31.3 – 66.5), NA = 2 |
| **Sex [Percent; (N)]** |  |
| Female | 37.5% (15) |
| Male | 57.5% (23) |
| NA | 5.0% (2) |
| **Hospitalizations [Percent; (N)]** | 40.0% (16), NA = 3 |
| **Vaccinated for Influenza [Percent; (N)]** | 32.5 (13), NA = 3 |
| **Comorbidities [Percent; (N)]** |  |
| Diabetes | 5.0% (2), NA = 3 |
| Chronic neurological disease | 2.5% (1), NA = 3 |
| Chronic renal failure | 0% (0), NA = 4 |
| Ischemic heart disease | 10.0% (4), NA = 3 |
| COPD | 20.0% (8), NA = 3 |
| Previous Stroke | 0% (0), NA = 3 |
| Smoker |  |
| Current | 40.0% (16) |
| Ex | 10.0% (4) |
| Never | 37.5% (15) |
| E-cigarettes | 2.5% (1) |
| NA | 10.0% (4) |
| Alcohol consumption | 10.0% (4), NA = 4 |
| **Fever [Percent; (N)]** | 92.5% (37), NA = 3 |
| **Fever Temperature [Median (IQR)]** | 38.5°C (38.2°C - 39°C), NA = 3 |
| **Nose Symptoms [Percent; (N)]** | 40.0% (16), NA = 3 |
| **Throat Symptoms [Percent; (N)]** | 37.5% (15), NA = 3 |
| **Headache [Percent; (N)]** | 45.0% (18), NA = 3 |
| **Nausea/Vomiting [Percent; (N)]** | 20.0% (8), NA = 3 |
| **Weakness [Percent; (N)]** | 82.5% (33), NA = 3 |
| **Arthralgia [Percent; (N)]** | 57.5% (23) NA = 3 |
| **Myalgia [Percent; (N)]** | 55.0% (22), NA = 3 |

**Supplementary Table 4. Marburg COVID-19 negative cohort clinical characteristics.**

|  | **COVID-19 Negative Patients (N = 19)** |
| --- | --- |
| **Age [Median (IQR)]** | 70 (57 – 81) |
| **Sex [Percent; (N)]** |  |
| Female | 47.4% (9) |
| Male | 52.6% (10) |
| **BMI [Median (IQR)]** | 24.0 (22.1 – 33.7) |
| **Comorbidities [Percent; (N)]** |  |
| Diabetes | 10.5% (2) |
| Obesity | 21.1% (4) |
| Chronic cardiac disease | 52.6% (10) |
| Coronary heart disease | 31.6% (6) |
| Arterial occlusive disease | 10.5% (2) |
| COPD | 15.8% (3) |
| Asthma | 0% (0) |
| Renal insufficiency | 36.8% (7) |
| Rheumatic disorders | 5.3% (1) |
| Neuromuscular disorders | 31.6% (6) |
| Oncological disorders | 21.1% (4) |
| Other immunosuppression | 15.8% (3) |
| **Duration of hospital stay [Median; IQR]** | 14.0 (8.0 – 23.0) |
| **Mortality [Percent; (N)]** | 5.3% (1) |
| **Intensive Care Unit [Percent; (N)]** | 52.6% (10) |
| Mechanical ventilation | 21.1% (4) |
| Supplemental oxygen | 63.2% (12) |
| **Therapy [Percent; (N)]** |  |
| antibiotics | 84.2% (16) |
| antivirals | 21.1% (4) |
| antimycotics | 5.3% (1) |
| Dialysis | 10.5% (2) |
| Extracorporeal membrane oxygenation | 0% (0) |
|  |  |

**Supplementary Table 5. Marburg Influenza patient cohort clinical characteristics.**

|  | Influenza Patients (n = 25) |
| --- | --- |
| Age [Median (IQR)] | 69.8 (56.5 – 81.5) |
| Sex [Percent; (N)] |  |
| Female | 52.0% (13) |
| Male | 48.0% (12) |
| BMI [Median (IQR)] | 28.2 (23.8-33.9), NA = 3 |
| CRP (mg/dl) | 124.3 (52.4 – 203.6) |
| Hospital Mortality [Percent; (N)] |  |
| Yes | 0% (0) |
| No | 88.0% (22) |
| NA | 12.0% (3) |
| Mechanical Ventilation [Percent; (N)] | 27.8 (23.7 – 33.3), NA = 3 |
| Yes | 4.0% (1) |
| No | 84.0% (21) |
| Non-Invasive Vent (NIV) | 4.0% (1) |
| NA | 8.0% (2) |
| Days Since First Symptoms [Median (IQR)] | 6.1 (2 – 6.8), NA = 1 |
| Pack Years [Median (IQR)] | 22.5 (0 – 40), NA = 3 |

**Supplementary Table 6. Giessen pneumonia-induced ARDS patient cohort clinical characteristics.**

|  | ARDS Patients (n = 17) |
| --- | --- |
| Age [Median (IQR)] | 56 (50 – 64.5) |
| Sex [Percent; (N)] |  |
| Female | 29.4% (5) |
| Male | 70.6% (12) |
| Secondary Diagnosis [Percent; (N)] |  |
| Viral pneumonia only (pathogen detected) | 52.9% (9) |
| Bacterial pneumonia only (pathogen detected)  Bacterial pneumonia only (pathogen not detected) | 11.8% (2)    17.6% (3) |
| Viral pneumonia with bacterial superinfection (pathogens detected) | 5.9% (1) |
| Not Known | 5.9% (1) |
| NA | 5.9% (1) |

**Supplementary Table 7. Traditional Autoantigen Array content.**

| **Bead ID** | **Sample** | **Vendor** | **Catalog #** |
| --- | --- | --- | --- |
| 1 | Bare Bead |  |  |
| 2 | Human IgG from serum | Sigma | I4506 |
| 3 | Anti-Human IgG Fc fragment specific | Jackson | 109-005-008 |
| 4 | Anti-Human IgG (H+L) | Jackson | 109-005-003 |
| 5 | Anti-Human IgG F(ab') fragment specific | Jackson | 109-005-006 |
| 6 | Beta 2 Glycoprotein 1 | Diarect | A14901 |
| 7 | Myeloperoxidase | Diarect | A18501 |
| 8 | La/SSB | Diarect | A12801 |
| 10 | Proteinase 3 | Diarect | A18601 |
| 11 | Histone 1 | Immunovision | HIS-1001 |
| 12 | Histone 2A and 4 | Immunovision | HIS-1002 |
| 13 | Histone 2B | Immunovision | HIS-1003 |
| 14 | CENP B | Diarect | A12501 |
| 15 | Histone 3 | Immunovision | HIS-1004 |
| 16 | Histones | Immunovision | HIS-1000 |
| 17 | GBM | Diarect | A16801 |
| 18 | C1q | Biodesign | A90150H |
| 19 | BPI | Arotec | ATB01-02 |
| 24 | Fibrillarin | Prospec | ENZ-566 |
| 26 | IA2 |  |  |
| 27 | GAD65 |  |  |
| 31 | U11/U12 | Origene | TP303746 |
| 38 | CENP A | Diarect | A16901 |
| 39 | EJ | Diarect | A11101 |
| 40 | HSP 70 | Stressgen | NSP-555 |
| 41 | HSP 90 | Stressgen | SPP-770 |
| 42 | Intrinsic Factor | Diarect | A16701 |
| 43 | JO1 | Diarect | A12901 |
| 44 | Ku, p70/p80 | Diarect | A17301 |
| 45 | LKM1 | Diarect | A13501 |
| 46 | MDA5 | Diarect | A30001 |
| 47 | MI-2 | Diarect | A18101 |
| 48 | PCNA | Diarect | A15401 |
| 49 | PL-12 | Diarect | A15701 |
| 50 | PL-7 | Diarect | A15601 |
| 51 | PM/Scl-75 | Diarect | A17001 |
| 52 | Nucleolin | Diarect | A19701 |
| 53 | Ribo P0 | Diarect | A14101 |
| 54 | Ribo P1 | Diarect | A14201 |
| 55 | PDC-E2 | Diarect | A17901 |
| 56 | Ribo P2 | Diarect | A14301 |
| 57 | SRP54 | Diarect | A18401 |
| 58 | PM/Scl-100 | Diarect | A16001 |
| 59 | POLR3H | Origene | TP310633 |
| 60 | PDH | Sigma | P7032 |
| 62 | Ro60 | Diarect | A17401 |
| 65 | Scl-70 | Diarect | A12401 |
| 68 | Smith | Immunovision | SMA-3000 |
| 70 | Troponin I | Prospec | PRO-1269 |
| 73 | TG | Diarect | A12201 |
| 74 | GNAL | Abnova | H00002774-P01 |
| 75 | MYH6 | Origene | TP313673 |
| 76 | TPO | Diarect | A12101 |
| 77 | ZnT8 |  |  |
| 79 | U1-snRNP A | Diarect | A13101 |
| 80 | U1-snRNP C | Diarect | A13201 |
| 83 | RPP25 (Th/To) | Origene | TP303538 |
| 84 | Insulin | Sigma | I0908 |

**Supplementary Table 8. pSTAT Induction Blocking Assay Conditions.**

| Cytokine | Cell Line | Concentration | Stim. Time  (min) | Target | Cell Line Preparation |
| --- | --- | --- | --- | --- | --- |
| IFNα2 | U937 | 2.5 ng/mL | 15 | pSTAT1 (Y701) | None |
| IFNα7 | U937 | 1000 U/mL | 15 | pSTAT1 (Y701) | None |
| IFNα8 | U937 | 2.5 ng/mL | 15 | pSTAT1 (Y701) | None |
| IFNγ | U937 | 2.5 ng/mL | 15 | pSTAT1 (Y701) | None |
| GM-CSF | U937 | 2.5 ng/mL | 30 | pSTAT5 (Y694) | None |
| IL-6 | THP-1 | 1 ng/mL | 20 | pSTAT3 (Y705) | None |

**Supplementary Table 9. pSTAT Induction Blocking Assay Cytokines and Antibodies.**

| **REAGENT** | **VENDOR** | **CATALOGUE #** |
| --- | --- | --- |
| **Cytokines** | | |
| IFNα2 | HumanKine | HZ-1066 |
| IFNα7 | PBL Assay Science | 111601 |
| IFNα8 | SinoBiological | 10347-H08H |
| IFNγ | HumanKine | HZ-1301 |
| GM-CSF | HumanKine | GM-CSF |
| IL-6 | HumanKine | HZ-1019 |
| **Blocking Antibodies** | | |
| anti-GM-CSF | R&D | AF215NA |
| anti-IL-6 | BioLegend | 501125 |
| **Staining Antibodies** | | |
| PE anti-STAT1 Phospho (Tyr701) | BioLegend | 666404 |
| PE anti-STAT3 Phospho (Tyr705) | BioLegend | 651004 |
| PE anti-STAT5 Phospho (Tyr694) | BioLegend | 936904 |
| PE Mouse IgG1k Isotype | BioLegend | 400139 |
| PE Rabbit IgG Isotype | Cell Signaling Technology | 5742S |

**Supplementary Table 10. GFP Reporter Blocking Assay Cytokines and Antibodies.**

| **REAGENT** | **VENDOR** | **CATALOGUE #** |
| --- | --- | --- |
| **Cytokines** | | |
| IFNα2 | BioLegend | 592704 |
| IFNγ | R&D Systems | 285-IF-100/CF |
| IFNλ2 | R&D Systems | 1587-IL-025/CF |
| IFNλ3 | R&D Systems | 5259-IL-025/CF |
| **Monoclonal Antibodies** | | |
| anti-IFNα2 | Invivogen | mabg-hifna-3 |
| anti-IFNγ | Invivogen | maba-hifng-3 |
| anti-IFNλ2 | Invivogen | mabg-hil28a-3 |
| anti-IFNλ3 | Invivogen | mabg-hil28b-3 |
